# Biodegradable Polylactide Nanocapsules Containing Quercetin for In Vitro Suppression of Mouse B16F10 and Human Sk-Mel-28 Melanoma Cell Lines

**DOI:** 10.3390/ph18070980

**Published:** 2025-06-30

**Authors:** Chenhui Zhao, Thomas Ming Swi Chang

**Affiliations:** Artificial Cells and Organs Research Centre, Departments of Physiology, Medicine and Biomedical Engineering, Faculty of Medicine and Health Sciences, McGill University, Montreal, QC H3G 1Y6, Canada; chenhui.zhao@mail.mcgill.ca

**Keywords:** quercetin, O-quinone, nanocapsules, nanobiotechnology, prodrug, melanoma, cancer, drug encapsulation, bioavailability

## Abstract

**Background:** Quercetin is a flavonoid found in various dietary sources. It is a prodrug converted by overexpressed tyrosinase in melanoma into an active o-quinone that suppresses tumour growth. However, injected quercetin is rapidly cleared from the tumour site. **Method:** Our study aimed to enhance quercetin’s efficacy through nanoencapsulation using biodegradable nanocapsules, which were tested in both mouse and human melanoma cell lines in 2D and 3D models. **Results:** Nanoencapsulation achieved sustained release and improved bioavailability. In mouse 2D cultures, quercetin nanocapsules (Q-nanos) reduced cell viability to 28%, compared with 46% for free quercetin (Q-only) (*p* < 0.05). In 3D cultures simulating in vivo conditions, Q-nanos reduced viability to 43%, showing significant anti-melanoma activity, while Q-only resulted in 72% viability (*p* > 0.05 vs. control). A similar trend was observed in human melanotic melanoma, where both Q-nanos and Q-only were effective compared with the controls, with Q-nanos demonstrating superior tumour inhibition (*p* < 0.05). **Conclusions:** These findings show the superior efficacy of nanoencapsulated quercetin over free quercetin. Nanoencapsulation prolonged quercetin’s bioavailability, enhanced tumour regression, and addressed limitations associated with the rapid clearance of free quercetin.

## 1. Introduction

Quercetin is a flavonoid found in various dietary sources that is primarily known for its antioxidant properties that include countering reactive oxygen species (ROS) and potentially averting cellular DNA damage [1,2]. When activated by the tyrosinase enzyme within these cells, quercetin oxidizes into reactive o-quinone and initiates the NQO1 and p53 pathways. The p53 pathway, which is intrinsically apoptotic, activates upon increased p53 protein levels. This triggers the transcription of apoptotic genes, elevating ROS levels and leading to endoplasmic reticulum stress-mediated apoptosis, particularly in highly tyrosinase-expressing cells such as those in melanoma. The crucial role of NQO1 involves stabilizing p53, preventing ubiquitin-independent p53 degradation, and recycling activated quercetin [3,4]. Unlike other treatments, quercetin induces apoptosis specifically in tumour cells, potentially eliminating tumours rather than solely suppressing their growth.

Melanoma is an aggressive skin cancer originating from melanocytes and is notorious for its invasive nature and metastatic properties. While it commonly emerges on the skin, it can manifest in various sites such as the mouth, intestines, or eyes. DNA mutations triggered by UV light exposure from the sun or tanning devices are the most common cause of melanoma. This cancer is highly prevalent, affecting approximately 3.1 million individuals worldwide. The increase in melanoma cases is closely linked to the ongoing depletion of the ozone layer [5,6].

A diverse range of melanoma treatments currently exists, each carrying its own set of advantages and limitations. While high-energy radiation effectively targets and destroys cancer cells, it also induces harm and skin-related issues. Surgical interventions offer a spectrum of options, from excising superficial tumours to extreme cases requiring amputation. Gene therapies and immunotherapies have shown effectiveness, though they come with undesirable side effects. These treatments often aim for tumour suppression or palliative care, bearing the risk of melanoma recurrence [5,6]. Hence, there is an urgent need for innovative and transformative therapies to revolutionize melanoma treatment [7,8]. Quercetin, while promising for melanoma treatment, faces limitations in stability, rapid breakdown, and low bioavailability in aqueous environments [9,10]. Its fast elimination from the bloodstream imposes challenges for effective reactivity, necessitating frequent doses for patients. These constraints have led to an urgent need for innovative strategies to optimize quercetin’s efficacy in melanoma therapy. Our research directly targets these delivery inefficiencies.

The development of biodegradable nanocapsules has led to the widespread use of nanocarriers in cancer therapy [7,8,11,12,13]. Our study employed biodegradable nanocapsules as carriers for quercetin. This extended the duration of drug release, as demonstrated by the release rate and kinetics. Such prolonged, controlled release holds promise compared with the potential adverse effects of conventional high-dose quercetin administration. Achieving sustained, low-dose release assists in mitigating these risks [12,13].

We selected polymeric poly-d,l-lactic acid (PLA) nanocapsules to achieve long-term delivery, as they are well suited for this purpose. Various nanodelivery systems exist, such as PLA, liposomes, chitosan carriers, and metallic nanoparticles, each with unique advantages. PLA nanocapsules, composed of biodegradable synthetic polymer that degrades into lactic acid, provide strong physical stability and sustained release [7,11]. Liposomes formed from natural phospholipid bilayers represent another popular method of drug nanoencapsulation [14]. Another method involves the use of chitosan-based carriers, which are derived from natural polysaccharides [15]. Metallic nanoparticles offer properties such as magnetism, optical activity, or photothermal effects, which are valuable for imaging and targeting [16]. We recently investigated nanorobotic superparamagnetic PLA nanocapsules that can be directed by an external magnetic field for targeted drug delivery [17]. Another approach is the use of thermo-responsive PNIPAAM-b-PLA amphiphilic block copolymer micelle as a nanoplatform for docetaxel drug release [18].

Expanding upon this foundation, we optimized the physical attributes of PLA nanocapsules and studied their release profiles under varied conditions. Our research indicated anti-cancer outcomes. Using 2D cell cultures, we observed evidence of reduced cell viability in the B16F10 melanoma cell line, supporting the effectiveness of our strategy involving nanocapsules loaded with quercetin. To enhance the reliability of our 2D cell culture findings, we transitioned to a 3D culture model. This strategic modification allowed us to establish a better connection between our results and potential clinical applications. Importantly, we expanded our investigation to compare the effects of quercetin nanocapsules (Q-nanos) with free quercetin (Q-only) solutions in this 3D culture using human melanoma cell lines, according to Patel et al. [19]. This allowed even more comprehensive evaluation of their therapeutic potential in humans.

In summary, this study demonstrates the use of polymeric nanocapsules for potential applications in cancer therapy. Employing biodegradable nanocapsules for quercetin delivery enabled us to achieve prolonged and controlled drug release. The observed reduction in cell viability in our 2D cell culture experiments supports the potential clinical significance of this approach. Furthermore, transitioning to the in vivo mimicking 3D culture model further supported our findings from the 2D experiments. The effectiveness of quercetin nanocapsules on human melanoma cell lines further shows the clinical potential of this research.

## 2. Results

### 2.1. Part 1—Nanocapsule Preparation and Properties

#### 2.1.1. Formation of PLA Nanocapsules Containing Quercetin Using Nanoprecipitation and Solvent Evaporation

**Figure 1:**
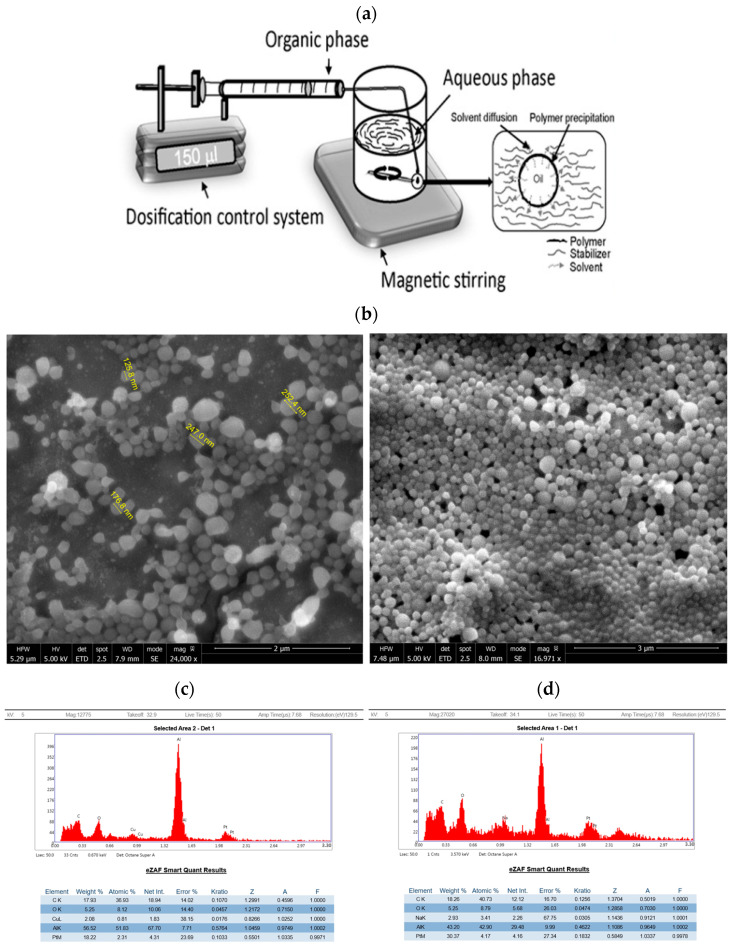

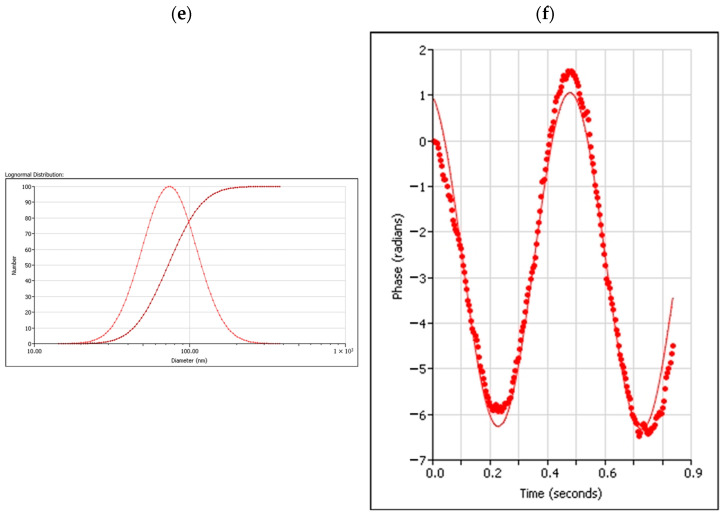
Experimental setup and physical characteristics of quercetin nanocapsules. (**a**): Experimental setup of the nanoprecipitation method. (**b**): SEM images showing close-ups of the quercetin nanocapsules and their size. (**c**): EDS analysis of empty nanocapsules. (**d**): EDS analysis of quercetin nanocapsules. (**e**): DLS size distribution of quercetin nanocapsules at a count rate (kcps) of 521.4 with a baseline index of 8.2. (**f**): Zeta potential distribution of quercetin nanocapsules at 683 μS conductance, 4.00 V voltage, 640 nm wavelength, and 2.00 Hz field frequency.

Quercetin-loaded nanocapsules (Q-nanos) were synthesized using the nanoprecipitation technique followed by solvent evaporation (Figure 1a). Successful synthesis yielded spherical nanocapsules displaying an orange hue due to quercetin encapsulation. The control nanocapsules lacking quercetin showed a circular shape and were colourless. Functionally, smaller nanocapsules can enter melanoma cells, while larger ones can accumulate in the surrounding microenvironment [7]. The nanocapsules showed spherical shapes and visibly contained quercetin. Varying sizes of nanocapsules can be prepared by adjusting the stirring speed; a speed of 700 rpm produced around 100 nm nanocapsules (Figure 1b). The EDS analysis comparing empty nanocapsules (Figure 1c) and quercetin nanocapsules (Figure 1d) showed that there were higher oxygen peaks in the quercetin nanocapsules, indicating the successful encapsulation of quercetin. The nanocapsules’ size distribution was further confirmed using dynamic light scattering (DLS). The prepared quercetin nanocapsules had an average diameter of 180.14 nm, polydispersity of 0.196 (Figure 1e), zeta potential of −20.87 mV, and mobility of −1.63 μ/s (Figure 1f).

#### 2.1.2. PH and Size Impact on Quercetin Nanocapsule Drug Release

**Figure 2:**
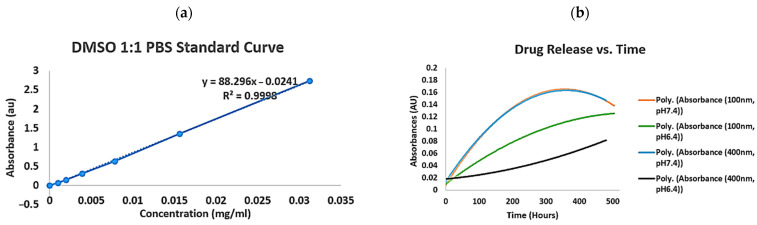
Quercetin nanocapsule release profiling. (**a**): Graph of quercetin standard curve in DMSO/PBS solutions. (**b**): Graph of drug release over time for different types of quercetin polylactic acid nanocapsules of different sizes (100 nm and 400 nm) in different pH environments (physiological pH 7.4; tumour acidic pH 6.4).

The drug release at pH 6.4 was 8.29 μg/mL/day for 100 nm nanocapsules and 4.57 μg/mL/day for 400 nm nanocapsules. At pH 7.4, the release rates were 8.39 μg/mL/day for 100 nm and 4.64 μg/mL/day for 400 nm nanocapsules (Figure 2). These findings indicate that the nanocapsules’ size significantly affected the drug release, with smaller nanocapsules (100 nm) exhibiting higher release rates compared with larger ones (400 nm). Moreover, the pH of the tumour environment also influenced the release rates, showing more release in higher pH environments regardless of size. This suggests that our PLA quercetin nanocapsules are more favourable for melanomas in higher pH environments. Additionally, smaller nanocapsules reached their peak release faster than larger ones.

### 2.2. Part 2—Effects of Quercetin Nanocapsules on the Mouse B16F10 Melanoma Cell Line

#### 2.2.1. Cell Viability Assay

**Figure 3:**
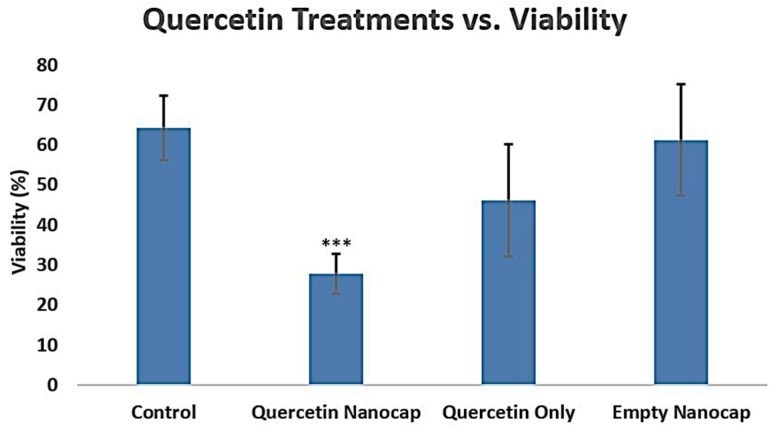
Graph of the 2D cell viability results on B16F10 melanoma cells. Control = distilled water; quercetin nanocap = quercetin-loaded nanocapsules (1770 µg/mL quercetin equivalent); quercetin only = free quercetin solutions (1770 µg/mL); and empty nanocap = empty polylactic acid (PLA) nanocapsules. *** indicates *p* < 0.05 compared to the control group.

After exposing cells to various treatments, they were cultured for three days with n = 6, followed by a viability assessment using a hemocytometer. The control groups and E-nano groups exhibited cell viability values within the 60–70% range, represented as mean ± s.e.m. At 1770 µg/mL quercetin equivalent, Q-nanos displayed a viability of 28%, while Q-only showed a viability of 46% in comparison, *p* < 0.05 (Figure 3). These results highlight distinct differences in cell viability between the Q-only and Q-nano groups compared with the controls, demonstrating the efficacy of quercetin against melanoma.

#### 2.2.2. MTT Assay

**Figure 4:**
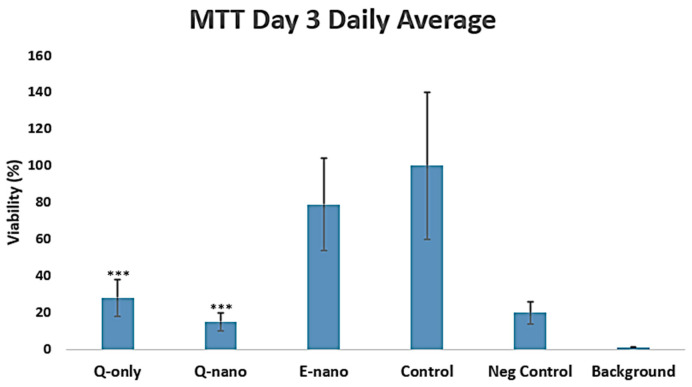
Graph of the average daily viability constructed from absorbance on day 3 using MTT assay. Q-only = free quercetin solutions; Q-nano = quercetin-loaded nanocapsules; E-nano = empty nanocapsules; Control = distilled water; Neg Control = MTT with DMEM alone; Background = empty background. All treatments were conducted with 1770 µg/mL quercetin equivalent. *** indicates *p* < 0.05 compared to the control group.

The results showed decreasing trends in both the Q-nano and Q-only groups across days 1, 2, and 3 compared with the control groups. However, no significant differences were observed in the viability between the Q-nano and Q-only groups on days 1 and 2. However, on day 3, the Q-nano groups showed significantly lower viability compared with the Q-only groups, 15% vs. 27%, *p* < 0.05, suggesting higher efficacy of these nanocapsules over an extended period (Figure 4). Overall, the Q-nano groups consistently exhibited the least cell viability among all groups. These findings might have been due to the gradual release of the quercetin core from nanocapsules into the environment compared with the immediate release in the Q-only group. In real physiological conditions, quercetin has a short circulation time and is metabolized within a few hours, while quercetin nanocapsules can remain in the cellular matrix releasing drugs over an extended period. Therefore, these experiments may have overestimated the effects of the Q-only groups and underestimated the effects of the Q-nano groups.

#### 2.2.3. Scratch Test

**Figure 5:**
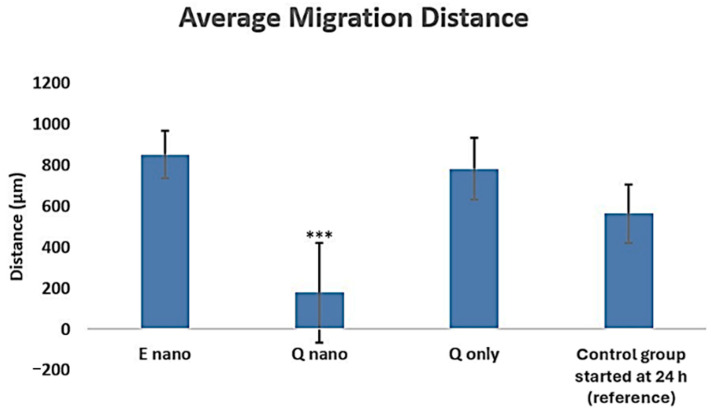
Graph indicating the average migration distances in both scratch tests. E-nano = empty nanocapsules; Q-nano = quercetin-loaded nanocapsules; Q-only = free quercetin solutions; and Control = distilled water group. After seeding each cell group onto 24-well plates and treating them with different drugs, a 24 h growth period was allowed. After that, the 2D culture received three washes with 1× PBS, followed by the addition of fresh cell culture media to each group. Using a sterile tip, wounds were created by scratching the cells. All treatments were conducted with 1770 µg/mL quercetin equivalent. *** indicates *p* < 0.05 compared to the control group.

After seeding each cell group onto 24-well plates and treating them with different drugs, a 24 h growth period was allowed. After that, the 2D culture received three washes with 1× PBS, followed by the addition of fresh cell culture media to each group. Using a sterile tip, wounds were created by scratching the cells. This procedure imitated the washing away of injected material by the circulating blood. The migration in the free quercetin group increased to 700 µm, whereas that in the quercetin nanocapsule group remained low (Figure 5). It is likely that the free quercetin was rinsed away by the multiple washings, while the quercetin nanocapsules continued to exert an effect. This could have been due to the entrapment of the nanocapsules in the cell culture, with some entering the cells.

#### 2.2.4. Colony Test

**Figure 6:**
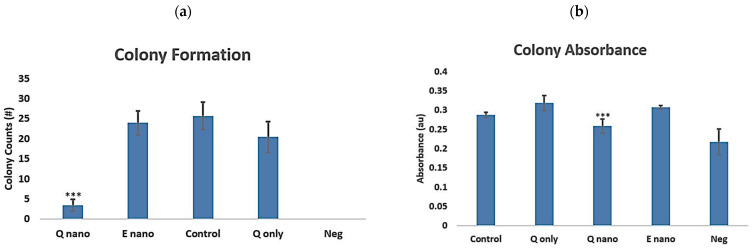
Graphs showing the colony formation ability of B16F10 melanoma cells under different treatments. E-nano = empty nanocapsules; Q-nano = quercetin-loaded nanocapsules; Q-only = free quercetin solutions; Control = distilled water group; and Neg = negative control for background. All treatments were conducted with 1770 µg/mL quercetin equivalent. (**a**) Numbers of colonies formed. (**b**) The corresponding absorbance in response to different treatments. # indicates the “number of” colonies, *** indicates *p* < 0.05 compared to the control group.

The colony test aimed to evaluate tumour formation potential post-metastasis. According to the results, both the Q-nano (<5 colonies) and Q-only (~20 colonies) groups had fewer colonies compared with the E-nano and control groups. However, the Q-nano groups showed fewer colonies compared with Q-only (*p* < 0.05) (Figure 6a). Analysis of crystal violet staining absorbances from the colonies also proved the significantly lower absorbance levels in the Q-nano groups compared with the Q-only and control groups. (Figure 6b).

#### 2.2.5. Annexin PI Dual Staining

**Figure 7:**
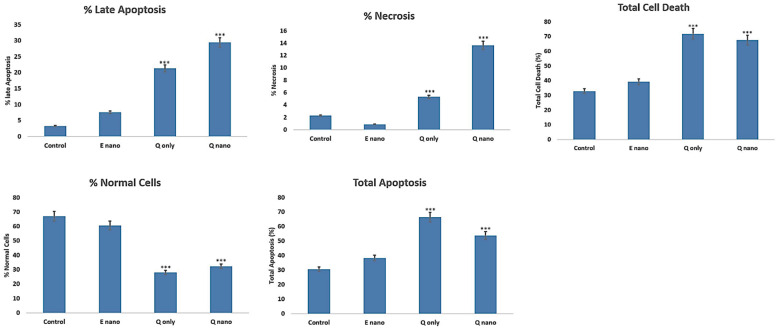
Graphs of the treatment results showing the percent of cells that stayed in the late apoptosis stage or necrosis stage, total cell death, and the total percent of normal cells remaining. E-nano = empty nanocapsules; Q-nano = quercetin-loaded nanocapsules; Q-only = free quercetin solutions; and Control = distilled water group. All treatments were conducted at 1770 µg/mL quercetin equivalent. *** indicates *p* < 0.05 compared to the control group.

The annexin V and PI dual-staining method is a sensitive approach for detecting cellular apoptosis (annexin V signal) and cellular necrosis or late apoptosis (PI signal). The absorbances measured via flow cytometry revealed clear outcomes. The Q-nano group showed higher signals in PI staining, indicating that most cells were in necrosis or late apoptosis stages compared with the control and Q-only groups (*p* < 0.05). In contrast, the Q-only groups showed more signals on annexin V staining, suggesting a higher occurrence of early apoptosis compared with the Q-nano groups (*p* < 0.05). Moreover, the Q-only groups showed more total apoptosis (late + early apoptosis), and total cell death (total apoptosis + necrosis) compared with the control groups (*p* < 0.05). However, these values were not significantly different compared with the Q-nano groups, indicating that both treatments induced a significant amount of apoptosis (Figure 7).

#### 2.2.6. Three-Dimensional Cell Culture Viability Study

**Figure 8:**
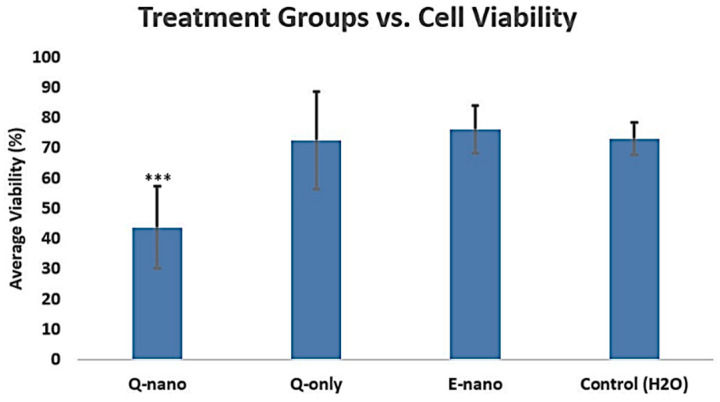
Graph showing the average percentage of cell viability in the 3D culture on day 7 after each treatment. Q-nano = quercetin-loaded nanocapsules; Q-only = free quercetin solutions; E-nano = empty nanocapsules; and Control (H_2_O) = distilled water group. All treatments were conducted with 1770 µg/mL quercetin equivalent. *** indicates *p* < 0.05 compared to the control group.

After 7 days, the Q-nano group showed the lowest cell viability (43%), which was lower than the Q-only group (72%) (Figure 8). The multiple washes and daily media changes removed free quercetins from the 3D gel, while quercetin nanocapsules were able to enter cells and exert cytotoxic effects. The *p*-value between the Q-only and control groups was calculated as 0.9546 (>0.05), indicating no significant differences in cell viability between them. However, there were significant differences between the quercetin nanocapsule and free quercetin groups. This highlights that under long-term in vivo conditions, free quercetin solutions are less effective in killing melanoma cells compared with quercetin nanocapsules.

#### 2.2.7. Multidose 3D Culture Study

**Figure 9:**
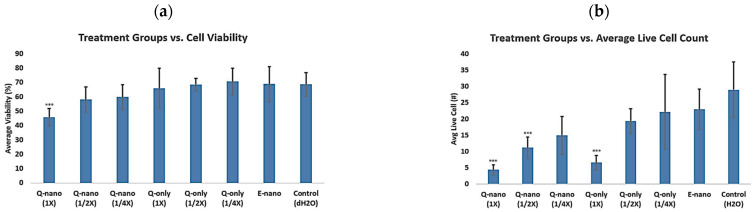
Graphs showing the results of multiple-dose testing in B16F10 melanoma cells. (**a**) Graph indicating the percent cell viability after different doses of treatments. Q-nano (1×, 1/2×, 1/4×) = quercetin-loaded nanocapsules at different concentrations; Q-only (1×, 1/2×, 1/4×) = free quercetin solutions at different concentrations; E-nano = empty nanocapsules; and Control (H_2_O) = distilled water group. 1× = 1770 µg/mL quercetin equivalent; 1/2× = 885 µg/mL; and 1/4× = 442 µg/mL. (**b**) Graph indicating the average live cell counts taken from 4 quadrants of the hemocytometer after different doses of treatments. # indicates “the number of” live cells, *** indicates *p* < 0.05 compared to the control group.

Figure 9a shows a clear trend in cell viability. The 1× Q-nano group showed the lowest cell viability at 45%, whereas the 1× Q-only group had a cell viability of 66%. This difference might have been due to the removal of free quercetins in the 3D gel through repeated washes, leaving the quercetin nanocapsules to target cells. Additionally, dose-dependent effects were evident, with 1/2× Q-nano at 58% and 1/4× Q-nano at 60% viability, which was notably more effective than 1× Q-only (65.9%). Comparatively, free quercetin, E-nano, and the water control groups showed no significant differences in viability (around 68–69%) on day 7 (Figure 9).

### 2.3. Part 3—Effects of Quercetin Nanocapsules in the Human Sk-Mel-28 Melanoma Line

#### 2.3.1. Two-Dimensional Cell Viability and Cell Count Assay

**Figure 10:**
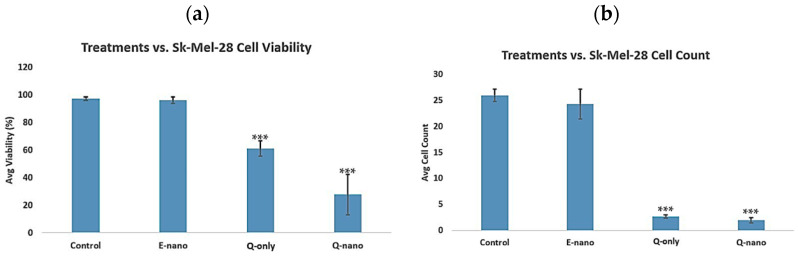
Two-dimensional cell viability test in the human melanoma Sk-Mel-28 line. E-nano = empty nanocapsules; Q-nano = quercetin-loaded nanocapsules; Q-only = free quercetin solutions; and Control = distilled water group. Q-nano = 1770 µg/mL quercetin equivalent. (**a**) Graph indicating the percent cell viability of treatments on the SK-Mel-28 line after 5 days of treatment. (**b**) Graph indicating the total cell counts taken from 4 quadrants of the hemocytometer after 5 days of treatment. *** indicates *p* < 0.05 compared to the control group.

After exposing cells to various treatments, they were cultured for five days, n = 5, followed by assessments using a hemocytometer. The control groups and E-nano groups exhibited high cell viability within the 96% range (Figure 10a) and high cell counts (Figure 10b) represented by the mean ± s.e.m. Q-nanos displayed a viability of 27% ± s.e.m. (*p* < 0.05 compared with the control), while Q-only showed a viability of 61% ± s.e.m. (*p* < 0.05 compared with the control). Quercetin nanocapsules and free quercetin were both effective against the Sk-Mel-28 line, as indicated by the reduced viability and cell counts. However, due to the limitations of 2D culture, the nanocapsules could not show their full advantages over free quercetin. Therefore, the *p*-values between Q-nanos and Q-only were 0.1 and 0.3, respectively, for the viability and cell counts.

#### 2.3.2. Three-Dimensional Cell Viability and Cell Count Assay

**Figure 11:**
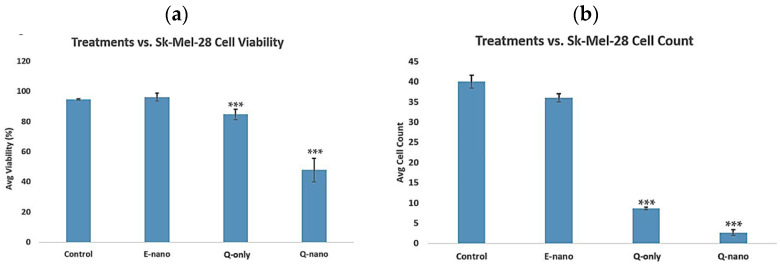
Three-dimensional cell viability test in the human melanoma Sk-Mel-28 line. E-nano = empty nanocapsules; Q-nano = quercetin-loaded nanocapsules; Q-only = free quercetin solutions; and Control = distilled water group. Q-nano = 1770 µg/mL quercetin equivalent. (**a**) Graph indicating the percent cell viability of treatments on the SK-Mel-28 line after 10 days of treatment on 3D collagen gel mix. (**b**) Graph indicating the total cell counts taken from 4 quadrants of the hemocytometer after 10 days of treatment. *** indicates *p* < 0.05 compared to the control group.

To improve the cell culture environment and change the cell culture medium to better mimic in vivo conditions, 3D culturing for 10 days was applied. After exposing cells to various treatments, they were cultured for 10 days, n = 5, followed by assessments using a hemocytometer. According to the results, the control and E-nano groups showed cell viability of around 95% (Figure 11a), and high cell counts (Figure 11b), represented as mean ± s.e.m. The Q-nano group showed a cell viability of 47% ± s.e.m. (*p* < 0.05 compared with the control), while the Q-only group showed a viability of 84% ± s.e.m. (*p* < 0.05 compared with the control). However, the *p*-values between the Q-nano and Q-only groups were 0.04 and 0.0001 for cell viability and cell counts, respectively. These results showed that in the 3D culture, the change of medium removed some free quercetin but left quercetin nanocapsules to affect the cells. The Q-only group had a low cell count, but its high cell viability indicated an initial tumour suppression burst effect; however, following medium removal, the cell viability was restored. This time, a significant difference was observed between the Q-only and Q-nano groups *p* < 0.05, showing that the Q-nano groups had a more significant effect on tumours in a 3D in vivo mimicking context. The Q-only groups still had *p* < 0.05 compared with the control groups, indicating the effectiveness of quercetin against this human melanoma cell line.

## 3. Discussion

Quercetin is a prodrug that is converted into its active form, o-quinine, by cancer cells with high expression of tyrosinase. However, it is rapidly removed after injection and thus requires large doses to cause toxic effects. Therefore, we studied the use of PLA nanocapsules to allow longer sustained release of quercetin (Figure 1). For over two decades, PLA has been used extensively in the context of implantation [8,11,20,21,22,23]. It is an FDA-approved biodegradable polymer that has been widely used in biomedical applications [24]. It degrades through hydrolysis into lactic acid, which is further metabolized into carbon dioxide and water. We previously studied the safety of PLA nanocapsules [12]. Although generally considered safe, local factors such as foreign-body reactions may still occur [25]. The encapsulation efficiencies of the formed PLA quercetin nanocapsules were 46% and 49% (Table 1). Several factors can contribute to efficient encapsulation, such as the solubility of quercetin in the organic phase and polymer–drug interactions. To improve encapsulation efficiency, we conducted a series of preliminary tests adjusting multiple parameters, such as the amount of PLA, stirring speed, and solvent evaporation time. The present study compares only the bioavailability of free quercetin with the PLA nanoencapsulated form. Having demonstrated this, the next step will be to optimize the encapsulation efficiency; for example, by adjusting PLA’s molecular weight, using copolymers such as PEG-PLA or co-solvents, and adjusting aqueous–organic phase ratios and surfactant concentrations.

After generating quercetin nanocapsules (Figure 1), our experiments showed a prolonged and controlled release of quercetin over several months, compared with its rapid clearance without nanocapsules within a few hours. This sustained release period, lasting months, provides the opportunity for quercetin to exert therapeutic effects, potentially leading to tumour regression. It enables a shift from frequent daily dosing to monthly injections. Different nanocapsules were also generated with various release rates (Figure 2), thus offering better treatment approaches based on the patient’s needs, disease stage, and urgency. Intravenous injections minimize gastrointestinal tract interactions, reducing potential side effects and drug resistance. The release profiles under different pH levels were also studied; in the quercetin PLA nanocapsules, a slightly higher quercetin release of 8.39 µg/mL/day was observed at pH 7.4 compared with 8.29 µg/mL/day at pH 6.4 (Figure 2) (Table 1). This may have been due to the way PLA degrades; hydrolysis can occur more easily at neutral pH because water penetrates the polymer more effectively, breaking it down faster. In contrast, mild acidity may slow down this process by reducing hydrolysis or slightly tightening the polymer structure. Although a slightly higher rate of release was observed at pH 7.4, this pH-related behaviour might be altered in vivo due to factors such as enzymatic activity, acidic tumour microenvironments, and cellular uptake mechanisms. This drug release profile could be improved by adjusting the composition and molecular weight of PLA polymers. Furthermore, incorporation of PEG-PLA copolymers could also be explored to enhance pH sensitivity, hydrophilicity, and circulation time, which we have also successfully developed as the next step of this research. Most importantly, the consistent, low-dose release mechanism lowers the risk of kidney damage associated with high-dose quercetin administration, offering a convenient and potentially cost-effective treatment modality [6].

Both the Q-nano and Q-only groups were effective in inducing cell stress, apoptosis, and necrosis (Figure 4 and Figure 7), and significantly reduced metastasis (Figure 5) and colony formation (Figure 6). In all cases, the Q-nano group showed a significantly stronger effect than free quercetin.

On days 1 and 2, the quercetin concentration from the Q-nano group may have remained below the cytotoxic threshold, whereas the Q-only group, being immediately bioavailable, produced an earlier effect. By day 3, the accumulated release from the Q-nanos reached therapeutic levels, leading to a significantly greater reduction in viability. This delayed but sustained release profile aligns with the nanocapsule design and indicates their potential for prolonged anti-tumour activity (Figure 4). The Q-nano group induced more necrosis/late apoptosis, while the Q-only group promoted early apoptosis. The Q-only group was rapidly removed, thus triggering only early apoptosis. This difference in pharmacokinetics may explain why the Q-nano group exerted a prolonged cytotoxic effect, while the Q-only group initiated only a transient apoptotic response.

These results indicate that the Q-nano and Q-only groups exhibited different pharmacokinetics. In vivo, rapid metabolism and the circulation of body fluids work to eliminate substances such as quercetin, posing a significant limitation to its use as a therapeutic agent due to rapid clearance [26]. In the 3D culture, nanocapsules were retained within the matrix, whereas the free quercetin was easily washed away, reflecting its rapid clearance in vivo. A major limitation of 2D cultures is the absence of a matrix to retain nanocapsules, making changes of medium difficult without removing nanoparticles before sufficient quercetin is released [27]. Consequently, the sustained presence and influx of free quercetin in 2D cultures may lead to overestimation of its anti-tumour effect, while underestimating the potential of quercetin nanocapsules.

To simulate human body conditions, we developed 3D cell cultures using rat tail collagen type I, a major extracellular matrix component [7]. Encapsulation within PLA nanocapsules emerged as an effective strategy to prevent rapid clearance. Even after continuous 5 h drug washes, quercetin-loaded nanocapsules reduced cell viability more than free quercetin (Figure 8). In a multidose study, Q-nano significantly reduced cell viability over 7 days compared with free quercetin. Notably, even a 1/4× dose of Q-nano was more effective than a 1× dose of free quercetin (Figure 9). Free quercetin showed no significant impact on cell viability after 7 days compared with the control groups. These findings demonstrate that nanoencapsulation prolongs quercetin’s bioavailability and enhances its anti-tumour efficacy in 3D culture.

To study clinical feasibility, we extended our investigation from mouse melanoma cells to the human Sk-Mel-28 melanoma line, evaluating efficacy in both 2D and 3D models. Sk-Mel-28 cells have distinct growth characteristics and morphology, proliferating slower than aggressive B16F10 cells. B16F10 cells rapidly form dense cell–cell interactions and expand quickly within the 3D matrix, whereas Sk-Mel-28 cells require a longer culture period to reach comparable cell density and 3D structure. To account for these differences, we adjusted the timeframes; 2D cultures were evaluated over 5 days and 3D cultures over 10 days, ensuring that both cell lines reached similar confluency for meaningful comparison. The results showed that both quercetin nanocapsules and free quercetin were effective in the 2D model, with no significant differences (Figure 10). However, in the 3D model, which better mimicked in vivo conditions, quercetin nanocapsules produced a significantly stronger anti-tumour effect than free quercetin, resulting in lower cell viability and cell counts (Figure 11).

The B16F10 melanoma cell line represents melanotic, high tyrosinase-expressing melanoma, mimicking classical pigmented, aggressive forms. In contrast, the Sk-Mel-28 human melanoma cell line represents amelanotic melanoma, with lower tyrosinase activity but retained enzymatic function, which is relevant to human melanomas characterized by low pigmentation [28]. This comparison covers different melanoma subtypes and enhances the translational value of our findings. Further studies on the use of quercetin nanocapsules in other high tyrosinase-expressing cancers, such as prostate tumours [29], are also warranted.

This study focused on prolonging the activity of quercetin through polymeric nanoencapsulation in both mouse and human melanoma cell lines, advancing the model from 2D to 3D. However, in vivo conditions may differ and could influence the performance of quercetin nanocapsules due to factors such as pH gradients, metabolism, and dynamic cell–cell interactions. Therefore, the next step is to conduct testing in more sophisticated models, such as organ-on-chip systems and in vivo animal studies, to confirm the clinical feasibility of quercetin nanocapsules.

## 4. Materials and Methods

### 4.1. Part 1—Nanocapsule Preparation and Properties

#### 4.1.1. Polylactic-(Dl)-Acid Nanocapsule Preparation

Empty nanocapsules, serving as controls, were prepared using the nanoprecipitation technique [7,8]. Initially, 90 mg of polylactic-(dl)-acid (PLA, ~20 kDa, Polyscience, Warrington, PA, USA) was dissolved in 3.2 mL of acetone (Sigma-Aldrich, St. Louis, MO, USA) and then mixed with 1.6 mL ethanol (Sigma-Aldrich, St. Louis, MO, USA) to enhance stability, forming an organic phase. The aqueous phase consisted of 10 mL of distilled water with 16 μL of Tween 20. The organic phase was introduced dropwise into the aqueous phase via a 26 G needle at a rate of 3mL/min. Stirring at room temperature favoured the formation of PLA polymer nanoparticles. After 1 h of stirring and solvent evaporation, the resulting PLA nanocapsules were then placed on microscope slides for size and morphology analysis.

#### 4.1.2. Quercetin Nanocapsule Preparation

An amount of 77.37 mg of quercetin (Abcam, Cambridge, UK, MW 302.236 g/mol) and 90 mg of PLA were dissolved in 3.2 mL of acetone to create a saturated solution. Precipitation led to a reduced amount of quercetin (38.5 mg), which was used in the quercetin–PLA organic phase. This organic phase was then gradually added dropwise into the aqueous phase using a 26 G needle at 3 mL/min while stirring at speed 2. Stirring continued at a speed of 700 rpm for 1 h in a well-ventilated environment for organic solvent evaporation, forming PLA nanocapsules around the quercetin molecules. The selection of this 700 rpm stirring speed was based on a series of preliminary experiments aimed at optimizing thew nanocapsules’ size and uniformity. Lower stirring speeds led to the formation of larger particles. By adjusting various parameters, including using specific surfactant concentrations, the resulting solution of quercetin nanocapsules [4.5 × 10^−4^ M] was stabilized with the addition of 150 μL Tween 20. Under these conditions, spherical nanocapsules 100–200 nm in diameter were prepared.

#### 4.1.3. Concentration and Purification

To concentrate the nanocapsules, the solvent elimination method was applied by evaporating ethanol and acetone from the organic phase via continuous stirring for an hour [7,8]. The purification of the nanocapsules was carried out using 100 kDa filters, effectively isolating the nanocapsules while draining out water and free quercetin. Following purification, the nanocapsules underwent three washes with distilled water to eliminate any remaining free quercetin molecules. Variations in the purification process allowed the preparation of different types of nanocapsules.

#### 4.1.4. Quercetin Nanocapsule Release at Different PH Levels

To monitor quercetin release over time at different PH levels, 3 mL of various quercetin micro/nanocapsules were introduced into 300 kDa dialysis bags placed in separate 60 mL PBS beakers at pH 7.4 or pH 6.4, simulating healthy or acidic tumour conditions [30]. The covered beakers were stirred and set in a Lab-Line Orbit Environ-Shaker (Kerala, India) at 90 rpm and 37 degrees to simulate movement. As the quercetin diffused from the nanocapsules, the released concentration was measured daily using a UV spectrometer.

### 4.2. Part 2—Effects of Quercetin Nanocapsules on Melanoma Cell Lines

#### 4.2.1. Tumour Cells and Culture Conditions

B16F10 murine melanoma cells (American Type Culture Collection ATCC, Manassas, VA, USA, #CRL-6475) and Sk-Mel-28 human melanoma cells (Creative Biogene, Inc., Shirley, NY, USA) were cultured in standard Dulbecco’s modified Eagle medium (DMEM) supplemented with 10% fetal bovine serum at 37 degrees and 5% CO_2_ in a humidified atmosphere.

#### 4.2.2. Two-Dimensional Cell Viability Assay

This study involved six treatment groups: quercetin-only (Q-only), quercetin nanocapsules (Q-nano), last wash of quercetin nanocapsules (Q-lw), empty nanocapsules (E-nano), last wash of empty nanocapsules (E-lw), and the control group (dH_2_O).

In the Q-lw and E-lw groups, the nanocapsules underwent three washes with distilled water via centrifugation at 11,000 rpm for 10 min each to eliminate cytotoxic Tween 20. The third washes of both the Q-nano and E-nano groups served as the “last-wash control”. Then, the nanocapsule solutions were vortexed at 2000 rpm/min to resuspend them. The resulting resuspended nanocapsule solutions were added to 10 mL cell culture dishes at a drug-to-cell ratio of 1:2. Following the application of different treatments, the cells were cultured for 3 days (n = 6), after which cell viability was assessed using a hemocytometer.

#### 4.2.3. MTT Assay

The six treatment groups (Q-only, Q-nano, Q-lw, E-nano, E-lw, and the control group (dH_2_O)) were prepared following the same setup as described previously for the cell viability study. Additionally, the negative control groups included MTT with DMEM alone and an empty background comparison. In 96-well plates, 100 μL of cells were seeded for each treatment group, divided into day 1, 2, and 3 subsets, n = 4 for each day. The cells were treated with drugs and left to settle overnight. Following incubation for 1, 2, and 3 days, the drugs were removed via 10× PBS washing. Subsequently, the cells were washed and lysed, and their absorbances were measured at 570 nm.

#### 4.2.4. Scratch Test

The cell migration test, commonly referred to as the scratch test, is a method for measuring metastasis. In this study, two types of scratch tests were conducted.

In the first group, after 24 h of cell culture in 24-well plates without drug treatment to facilitate complete growth and plate coverage, various treatments were applied. A sterile pipette tip was used to create a wound by scratching the plate.

For the second group, after seeding each cell group onto 24-well plates and treating them with different drugs, a 24 h growth period was allowed. After that, the drugs were removed through three washes with 1× PBS, followed by the addition of fresh drug-free cell culture media to each group. Wounds were created by scratching the cells with a sterile tip.

For both groups, the healing and migration of these wounds were tracked by measuring the wound width in micrometres (μm) at 0 h, 24 h, 40 h, and 49 h. The “Distance vs. Time” graph illustrates the changes in wound width over time.

#### 4.2.5. Colony Test

Six groups with n = 4 were assigned: empty nanocapsules (E-nano), quercetin only (Q-only), quercetin nanocapsules (Q-nano), quercetin last wash (Q-lw), the control (cells without drugs), and the negative control (only plate, no cells) serving as background. The cells were cultured in 24-well plates, subjected to different treatments, and incubated for 48 h. Post-incubation, the cells were collected and placed onto nutrient agar plates (1% agar base, 0.7% agar top) for a 3-week growth period. Subsequently, staining with crystal violet was conducted. The number of colonies formed was counted after three washes with 1× PBS. To release the staining from the colonies, cell lysis with DMSO was performed, and absorbance was measured after 24 h.

#### 4.2.6. Annexin PI Dual Staining

After three days of treatment, the cells were stained with binding buffer/annexin/PI and analyzed using the FACSCalibur flow cytometer (BD Biosciences, Franklin Lakes, USA). The PI-only and annexin-only groups served as the background controls.

#### 4.2.7. Three-Dimensional Cell Culture Viability Study

Six experimental groups were established: quercetin nanocapsules (Q-nano), quercetin solution (Q-only), quercetin nanocapsule last wash suspension (Q-nano-sus), empty nanocapsules (E-nano), empty nanocapsule last wash suspension (E-nano-sus), and the control (water). In 24-well plates, a collagen mix was added to each well and gently stirred to ensure fine mixing of cells, drugs, media, and collagen. The mixture was incubated to allow firm gel formation with final concentrations of 106.5 μL drug–cell mixture (35.5 μL treatment + 71 μL cells), 355 μL type I collagen, 50 μL 10× PBS buffer, and 8.5 μL 1N NaOH at a pH of 7. Following successful gel formation, PBS/media wash solutions were gently added for wash diffusion of the drugs, with each wash lasting 1 h for a total of 5 washes. After washing, fresh medium was added, and cells were incubated. Media changes were performed daily for 7 days to facilitate growth. After 7 days, the collagen gel matrix was digested using collagenase at a concentration of 4 mg/mL, and cell counts were conducted using a hemocytometer and trypan blue dye.

#### 4.2.8. Multidose 3D Culture Study

Eight experimental groups were established: quercetin nanocapsules (Q-nano) at concentrations of 1×, 1/2×, 1/4×; free quercetin solution (Q-only) at concentrations of 1×, 1/2×, 1/4×; empty nanocapsules (E-nano); and the control (water), with 1x stock solution being 1770 μg/mL. In 24-well plates, a collagen mix was added to each well and gently stirred to mix cells, drugs, media, and collagen. The mixture was incubated to allow firm gel formation. PBS/media wash solutions were added for 1 h per wash to allow drug diffusion, with a total of 5 washes. After washing, fresh media was added, and the cells were incubated. Media changes were performed daily for 7 days to support growth. After 7 days, the collagen gel matrix was digested using collagenase at a concentration of 4 mg/mL. Cell viability and total cell counts were determined using a hemocytometer and trypan blue dye.

#### 4.2.9. Data Analysis

Statistical analysis was performed using the Student’s *t*-test or one-way ANOVA and compared with the control group for significance at a *p*-value < 0.05. For experiments involving more than two groups, post hoc analysis was conducted using Tukey’s HSD test following one-way ANOVA.

## 5. Conclusions

In summary, our nanobiotechnological approach—specifically, nanoencapsulation—enabled the creation of biodegradable PLA quercetin nanocapsules. This study addressed the rapid clearance of the anti-cancer prodrug quercetin within the body, which is a major limitation that hinders its therapeutic application. The developed nanocapsules demonstrated prolonged quercetin release, increased bioavailability, and inhibited tumour growth in both 2D and 3D models of melanoma, showing their potential for application in high tyrosinase-expressing tumours. Our study findings suggest that nanoencapsulation could lead to more effective cancer treatments with reduced frequency of dosage and fewer side effects, demonstrating the relevance of the use of nanotechnology for medical applications. However, the current study also demonstrates the complexities of translating 2D to 3D and in vitro to in vivo outcomes. Therefore, there is a need for further research to fully understand the potential of quercetin nanocapsules in vivo and in clinical settings.

Overall, our study serves as a reference for future nanocarrier systems research and development for drug delivery, offering another approach towards the improvement of cancer treatment strategies.

## Figures and Tables

**Table 1:** Table summarizing the properties and release profiles of quercetin nanocapsules.

Samples	Quercetin PLA Nanocapsules (100 nm, 700 rpm)	Quercetin PLA Nanocapsules (400 nm, 400 rpm)
Encapsulation efficiency (%)	46%	49%
Peak release (μg/mL/day)	8.39 (pH 7.4 PBS) 8.29 (pH 6.4 PBS)	4.64 (pH 7.4 PBS) 4.57 (pH 6.4 PBS)
Time required to reach peak release (h)	288 (pH 7.4 PBS) 432 (pH 6.4 PBS)	360 (pH 7.4 PBS) 408 (pH 6.4 PBS)

## Data Availability

Data are available upon request from the authors.
